# The Roles of miRNA in Glioblastoma Tumor Cell Communication: Diplomatic and Aggressive Negotiations

**DOI:** 10.3390/ijms21061950

**Published:** 2020-03-12

**Authors:** Andrei Buruiană, Ștefan Ioan Florian, Alexandru Ioan Florian, Teodora-Larisa Timiș, Carmen Mihaela Mihu, Maria Miclăuș, Sergiu Oșan, Iona Hrapșa, Radu Constantin Cataniciu, Marius Farcaș, Sergiu Șușman

**Affiliations:** 1 Department of Medical Genetics, Iuliu Hațieganu University of Medicine and Pharmacy, 8 Victor Babes Street, 400012 Cluj-Napoca, Romania; andrei.buruiana@yahoo.com (A.B.); sergiuosan96@gmail.com (S.O.); i.hrapsa@gmail.com (I.H.); cataniciuradu@gmail.com (R.C.C.); marius.seraph@gmail.com (M.F.); 2 Department of Neurosurgery, Iuliu Hațieganu University of Medicine and Pharmacy, 8 Victor Babes Street, 400012 Cluj-Napoca, Romania; stefanfloriannch@gmail.com (Ș.I.F.); florian.ioan.alexandru@gmail.com (A.I.F.); 3 Department of Neurosurgery, Emergency County Hospital, 3-5 Clinicilor Street, 400006 Cluj-Napoca, Romania; 4 Department of Physiology, Iuliu Hațieganu University of Medicine and Pharmacy, 8 Victor Babes Street, 400012 Cluj-Napoca, Romania; doratimis@gmail.com; 5 Department of Morphological Sciences-Histology, Iuliu Hațieganu University of Medicine and Pharmacy, 8 Victor Babes Street, 400012 Cluj-Napoca, Romania; carmenmihu2004@yahoo.com; 6 Department of Medical Genetics, Emergency Hospital for Children, 68 Moților Street, 400370 Cluj-Napoca, Romania; m.miclaus@outlook.com; 7 Department of Genetics, IMOGEN Research Center, Louis Pasteur Street, 400349 Cluj-Napoca, Romania; 8 Department of Pathology, IMOGEN Research Center, Louis Pasteur Street, 400349 Cluj-Napoca, Romania

**Keywords:** glioblastoma, miRNA, intercellular communication, tumor microenvironment

## Abstract

Glioblastoma (GBM) consists of a heterogeneous collection of competing cellular clones which communicate with each other and with the tumor microenvironment (TME). MicroRNAs (miRNAs) present various exchange mechanisms: free miRNA, extracellular vesicles (EVs), or gap junctions (GJs). GBM cells transfer miR-4519 and miR-5096 to astrocytes through GJs. Oligodendrocytes located in the invasion front present high levels of miR-219-5p, miR-219-2-3p, and miR-338-3p, all related to their differentiation. There is a reciprocal exchange between GBM cells and endothelial cells (ECs) as miR-5096 promotes angiogenesis after being transferred into ECs, whereas miR-145-5p acts as a tumor suppressor. In glioma stem cells (GSCs), miR-1587 and miR-3620-5p increase the proliferation and miR-1587 inhibits the hormone receptor co-repressor-1 (*NCOR1*) after EVs transfers. GBM-derived EVs carry miR-21 and miR-451 that are up-taken by microglia and monocytes/macrophages, promoting their proliferation. Macrophages release EVs enriched in miR-21 that are transferred to glioma cells. This bidirectional miR-21 exchange increases STAT3 activity in GBM cells and macrophages, promoting invasion, proliferation, angiogenesis, and resistance to treatment. miR-1238 is upregulated in resistant GBM clones and their EVs, conferring resistance to adjacent cells via the CAV1/EGFR signaling pathway. Decrypting these mechanisms could lead to a better patient stratification and the development of novel target therapies.

## 1. Intercellular Communication—Friend or Foe?

Alongside proliferation and differentiation, intercellular communication represents one of the fundamental characteristics of multicellular organisms. The emergence of communication during evolution offered the possibility of developing increasingly complex supracellular structures, capable of achieving functions that allow adaptation to diverse conditions. 

The molecular mechanisms behind intercellular communication include soluble factors (cytokines/chemokines, growth factors, hormones, and neurotransmitters) and also direct exchange of miRNA via extracellular vesicles (EVs), or gap junctions (GJs). Numerous studies have shown that communication between tumoral cells and stromal/immune cells has many similarities to physiological communication, using the same mechanisms mentioned above. 

Nowadays, it is accepted that tumors have many effects upon the microenvironment, transforming the homeostatic conditions into a favorable medium for growth, invasion, and angiogenesis. In this context, transferred miRNA between cells get essential roles in the cell-cell communication through the modulation of the phenotype of neighboring and even distant cells.

## 2. Glioblastoma—A Whole-Brain Disease

Glioblastoma (GBM) is a WHO grade IV glioma, the most frequent and aggressive primary malignant brain tumor [1,2]. Some particularities are responsible for its aggressiveness, outlining a complex picture of histopathologic, molecular, and genetic elements. Epidermal growth factor receptor (*EGFR*) overexpression, *PTEN* (*MMAC I*) mutation, *CDKN2A* (p16) deletion, and less frequently *MDM2* amplification are just a few defects which lead to uncontrolled proliferation, invasion, and angiogenesis in primary glioblastoma (GBM) [3]. *TP53* mutations are usually the earliest genetic alterations detected in secondary GBM [3,4]. Unlike other gliomas, GBM has a unique histological pattern characterized by poorly differentiated neoplastic astrocytes that infiltrate widely, particularly along white matter tracts, and spread through the corpus callosum towards the other cerebral hemisphere [1]. The high proliferation rate demands an accelerated metabolism, creating hypoxic areas that trigger increased expression of VEGF. The large quantities of VEGF, along with hypoxia and the crosstalk between angiogenesis and proliferation, result in the pathognomonic elements of GBM: immature vascular proliferation and/or necrosis [5]. 

The current standard of care, surgical resection followed by temozolomide (TMZ) chemotherapy and radiotherapy, provides a median survival of only 14.6 months [6]. Unfortunately, almost all patients develop resistance to the standard treatment over time, leading to highly aggressive recurrences located 2–3 cm from the border of the original lesion [7]. The resistance to treatment arises from the intra-tumoral heterogeneity, a phenomenon generated by genetic mutations and, consequently, by phenotype adaptations, as well as by alterations of the cell-cell communication. Numerous subgroups formed by resistant clones occur pre- or post-exposure to treatment, driving to a multitude of cells with different molecular and behavioral characteristics [8,9]. A distinct subset of tumor cells, glioma stem-like cells (GSCs), possesses neural stem cells features and is responsible for self-renewal and soluble factors secretion but also chemo- and radio-resistance. Besides tumor cells, the GBM network consists of normal brain cells (astrocytes, microglia, endothelial cells, and neurons) and peripheral immune cells (monocytes/macrophages and lymphocytes), modeling a complex tumor microenvironment (TME). 

This review aims to present the key roles of miRNAs in the communication within the GBM microenvironment, underling both the intracellular function of modulating secretable factors and the intercellular transfer between different cell types.

## 3. MicroRNAs—Biogenesis and Roles in Glioblastoma Cells 

MicroRNAs are a class of non-coding, single-stranded RNA 21–25 nucleotides in length [10]. miRNAs play very important roles, being involved in the post-transcriptional regulation of gene expression. Currently, over 2000 microRNAs have been identified in humans. Genes for miRNAs are located in introns or exons, both in coding and non-coding transcription units, the majority of them being grouped in clusters [11]. 

**Figure 1:**
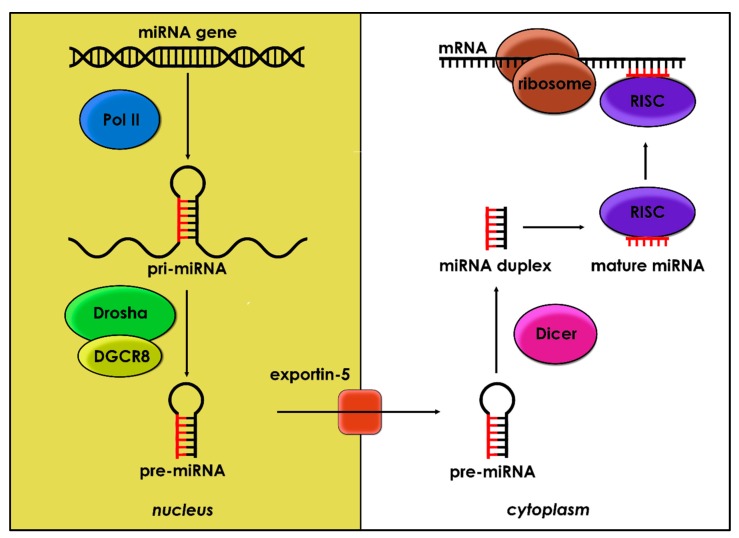
miRNA biogenesis. The red strand represents the guide strand and the black strand represents the passenger strand. Abbreviations: Pol II = polymerase II, pri-miRNA = primary miRNA, pre-miRNA = precursor miRNA, DGCR8 = DiGeorge syndrome critical region in gene 8, RISC = RNA-induced silencing complex.

miRNA genes are mostly transcribed by RNA polymerase II (Pol II) into long molecules (hundreds of nucleotides) as primary miRNA (pri-miRNA) [12]. Formerly, pri-miRNA is cleaved by the Drosha enzyme and its cofactor DiGeorge syndrome’s critical region in gene 8 (DGCR8), resulting in precursor miRNA (pre-miRNA), a 70–80 nucleotide stem-loop [13]. Pre-miRNA hairpin is then transported by exportin-5 from the nucleus into the cytoplasm, where the stem-loop is cleaved by RNase III enzyme Dicer, and a double-stranded miRNA emerges [14]. The miRNA:miRNA duplex is incorporated onto Argonaute protein 2 (Ago2) to form the RNA-induced silencing complex (RISC). Generally, one strand of miRNA remains as the mature miRNA (guide strand), while the other one (passenger strand) is degraded by Ago2 [15]. The guide strand recognizes the base-pairing complementary sequence of the target messenger RNA (mRNA), and RISC accomplishes RNA-silencing through cleavage or translation repression [16]. Due to the small length, each miRNA can silence several mRNAs, and each mRNA can be repressed by more than one miRNA (Figure 1).

In cancer, the miRNA expression is abnormal due to amplification, deletion, translocation, or epigenetic silencing of miRNA genes; the dysregulation of transcription factors (e.g., p53 and c-Myc); and defects in the biogenesis enzymatic equipment (e.g., point substitutions/deletions of *DGCR8* or *DROSHA*, mutations of *XPO5*—exportin encoding gene, and Dicer dysregulations) [17]. Upregulated miRNAs can act as oncogenes (oncomiRs) and silence the oncosuppressor genes, whereas downregulated miRNAs may function as tumor suppressors. A systematic review of miRNAs in GBM identified 253 overexpressed and 95 underexpressed miRNAs, while 17 miRNAs are controversially regulated [18]. 

The downregulated miRNAs earned extended recognition as possible therapeutic molecules. For example, the miR-7, miR-34a, miR-124, miR-128, miR-137, and miR-181 family are just a few underexpressed miRNAs whose forced expression inhibits GBM development [19,20]. Regarding elevated miRNAs, miR-21 was the first investigated oncomiR. miR-21 plays important roles in malignant processes by targeting genes involved in proliferation (*ANP32A*, *SMARCA4*, *PTEN*, *SPRY2*, and *LRRFIP1*); cell survival (*HNRPK, TAp63*, and *PDCD4);* invasion (*RECK* and *TIMP3*); and treatment resistance [18]. The miR-17-92 cluster (miR-17-3p, miR-17-5p, miR-18a, miR-19a, miR-19b, miR-20a, and miR-92a) is also overexpressed in GBM and promotes the silencing of antiproliferative genes (*TGFBRII*, *SMAD4*, and *CAMTA1*); regulators of angiogenesis; and apoptosis. Other upregulated miRNAs were intensively studied and found to play crucial functions in gliomagenesis (miR-10b, miR-15b, miR-26a, miR-93, miR-148, miR-182, and miR-221/222) [18]. 

On the other hand, miR-451 has debatable expressions and roles. Although in GBM cell lines miR-451 was showed to be downregulated and to function as a tumor suppressor, another study discovered that miR-451 is enhanced in GSCs [21]. However, these contradictory results can be explained by the fact that miR-451 is regulated as a metabolic adaptation. Hence, in hypoglycemic conditions, miR-451 is underexpressed, whereas normal glucose conditions increase miR-451. miR-451 overexpression is linked to the repression of CAB39/LIKB1/AMPK pathway, leading to mTOR activation and increased proliferation [22]. Conversely, CAB39/AMPK pathway activation promotes invasion and inhibits proliferation. This mechanism suggests a switch between proliferation and migration as GBM cells seek nutrients in order to progress.

## 4. Mechanisms of Cellular Communication via miRNA

**Figure 2:**
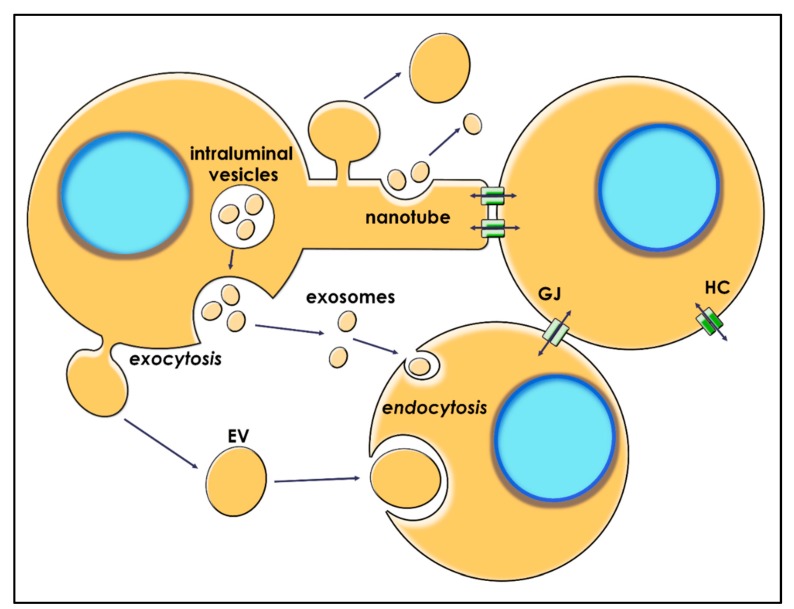
The mechanisms of intercellular communication via miRNA. miRNA can be released as Ago2-bound miRNA or packaged in membrane structures (exosomes and extracellular vesicles). Exosomes and extracellular vesicles (EVs) are englobed by recipient cells through endocytosis or pinocytosis, and miRNAs exert their effects at this level. Cell-cell contact can be mediated by gap junctions (GJs) which directly transfer miRNA between adjacent cells. A similar mechanism is realized by nanotubes which permit direct contact at a distance. Connexins from GJs may also form hemichannels (HCs) and ensure communication with the extracellular environment.

Numerous signaling pathways have been identified in GBM. Cytokines/chemokines secreted in the TME have been shown to contribute significantly to the recruitment of normal cells in order to support tumor growth, invasion, angiogenesis, and metastasis [23]. Still, other factors, especially miRNAs, have gained attention in recent years. Overexpressed miRNAs are present in high quantities in body fluids such as plasma and the cerebrospinal fluid (CSF), participating in intercellular communication and serving as potential prognostic and diagnostic biomarkers [24,25]. The exact mechanism of miRNAs release is not clear, but their presumed origin is both from apoptotic bodies and from active release by viable cells [26]. Still, some mechanisms of miRNA exchange, such as gap junctions, exosomes, extracellular vesicles, and tunneling nanotubes, were described in many tumors, including GBM (Figure 2).

### 4.1. Extracellular Ago2-Bound miRNAs

Two independent studies have shown that 90%–99% of miRNAs are located extracellularly, coupled with Ago2 protein [27,28]. By this association, miRNA escapes the action of nucleases in the body fluids. However, their actions are not specific, and these miRNAs are considered to be the result of physiological cellular activity [29]. Further research is needed to elucidate the precise role of Ago2-bound miRNAs in the intercellular communication and tumor progression.

### 4.2. Extracellular Vesicle miRNAs

Despite the majority of extracellular miRNAs being Ago2-bound, several quantities are found in EVs, this packaging also showing protective features [27]. EVs are double-layer phospholipid membrane vesicles which are released by all cells and represent a major component of intercellular communication, mediating the transfer of small molecules such as non-coding RNA (lncRNA and miRNA), messenger RNA (mRNA), DNA, proteins, and lipids. This process is influenced by various occurrences in the cell life cycle which may modify the general output, profile, load, and configuration of EVs, particularly the repertory of associated proteins, fatty acids, and RNAs [30,31]. Thus, the discharges of miRNA by EVs are selective and vary depending on the cell type, being packaged in specific structures that illustrate their origin. 

Based on origin and dimension, EVs can be classified as: ectosomes, exosomes, oncosomes, and microvesicles [32,33,34,35]. Exosomes (30–150 nm in diameter) derive from intraluminal vesicles that merge with the plasma membrane or are directly delivered in the microenvironment [36]. Larger than exosomes, microvesicles (100–1000 nm) develop from the cell membrane and are discharged by outward budding of the cell membrane [36].

Recent studies have illustrated the roles of EVs in tumor proliferation, invasion, and treatment resistance [30]. In GBM, dysregulated levels of miRNA were found in EVs, suggesting the alteration of intercellular communication [37,38]. In addition, more than 95% of miRNAs packaged in CSF EVs are also present in crude CSF, highlighting a balance of miRNA levels between these two compartments [39]. 

### 4.3. Gap Junctions and Hemichannels

GJs are formed by connexins and mediate direct cell-cell contact, establishing a network of interconnected cells. Through these connections, adjacent cells exchange ions, metabolites, ATP, DNA, and RNA that influence diverse cell functions [40]. It is supposed that miRNA transport through gap junctions is mediated by modifications in GJ permeability which control the distribution of intracellular miRNA [41,42,43]. Thus, miRNA transfer through GJs permits the spatial-temporal modulation of gene expression in neighboring cells, favoring cellular organization. 

Peng et al. demonstrated that only connexin43 (Cx43) GJs are permeable for 18–27 nucleotides miRNAs [44]. GBM cells, astrocytes, and endothelial cells express Cx43 and construct functional GJs, allowing for effective gap junction intercellular communication (GJIC) [45,46]. Pioneering studies in this field performed through immunohistochemistry (IHC), Western blot and Northern blot techniques have shown a negative correlation between Cx43 levels and the glioma grade, GBM having a low Cx43 expression [47,48,49,50]. More recently, Crespin et al. illustrated through tissue microarray that, while Cx43 distribution is heterogeneous in the tumor area, it is constantly detected at the periphery of vessels and astrocytes [51]. Hence, although global analysis by Western blot shows the low expression of Cx43 in GBM, in situ research highlights the presence of Cx43 in relation to astrocytes and endothelial cells. 

In addition to GJ, connexins can form hemichannels which mediate the miRNA transfer between cytoplasm and the extracellular environment in the same manner, allowing communication with distant cells [52,53].

### 4.4. Tunneling Nanotubes

Tunneling nanotubes (TNTs) are protrusions of the cellular membrane responsible for proximal and distant communication, creating multicellular anatomical networks. TNTs are crucial for embryonic development and normal functioning but may also act in cancer communication by GJIC formation and EVs release [54]. Along with small molecules and even organelles, miRNA can be transferred via TNTs. Osswald et al. showed that TNTs contribute significantly to shaping the glioma network, increasing resistance to treatment [55]. 

All these mechanisms are partly responsible for intercellular transfer between different cell populations within GBM and are briefly illustrated in Table 1. These processes will be explained in detail in the next sections.

## 5. Glioblastoma—Glial Cells Crosstalk

### 5.1. Astrocytes—Siding with The Enemy

Glioblastoma shapes a unique microenvironment in its proximity that favors tumor development, engaging healthy neighboring cells in this process. Since astrocytes represent the most abundant type of glial cells and also one of the cell types from which gliomas originate, there is bound to be a significant interaction between the two. As a response to CNS damage, astrocytes undergo a series of structural and functional changes, transforming into reactive astrocytes in order to repair the affected tissue. However, in the case of GBM, this process seems to be quickly diverted to support tumor development [63]. Tumor associated astrocytes were shown to be activated by GBM cells and further contribute to tumor growth, invasion, and resistance to treatment [64,65]. There seems to be a mutual exchange of signal-molecules through which glioma cells and astrocytes modify each other’s behavior, and numerous paracrine factors have already been described in other papers. As the contribution of miRNAs to GBM growth and invasiveness is increasingly explored, we will focus on their roles in the glioma cell-astrocyte communication.

In glioma-associated astrocytes, Cx43 is strongly upregulated, especially in the peritumoral region [50,60]. Regarding glioma-glioma gap junctions, Aftab at al. showed that knockdown of Cx43 in U118 cells by five distinct short hairpin RNAs (shRNA) resulted in a decrease in GJIC formation [66]. Using a wound-healing assay and also a 3D migration model (spheroid migration assay), an increase in glioma cells’ migration was depicted. Strale et al. showed that Cx43 repression by shRNA increased migration of U251 cells in a wound-healing assay, Boyden chamber, and in brain slices [67]. Analyzed together, all these data confirmed that glioma-glioma GJs have anti-invasive effects. 

Instead, glioma-astrocyte and astrocyte-astrocyte GJs were shown to promote glioma invasion. Sin et al. evidenced that mouse GL261 glioma cells formed circumscribed tumors in Cx43 conditional knockout mice, while tumors developed irregular borders of the invasion front in the wild-type animals [56]. The distance from the tumor periphery to brain regions lacking detectable GFAP immunoreactivity was measured, showing increased tumor-induced astrogliosis in Cx43-null mice in comparison with wild-type mice. These findings are supported by in vitro studies exploiting different cell lines. Zhang et al. co-cultured Cx43-transfected rat C6 glioma cells with rat astrocytes, the in vitro cell motility assay revealing that the invasive index of C6-Cx43 cells was significantly higher than that of C6 control cells [68]. More recently, Hong et al. used human cells (U87 GBM cells and human astrocytes) co-cultured in matrigel to simulate the TME and found a similar behavior of invasion [57]. 

**Figure 3:**
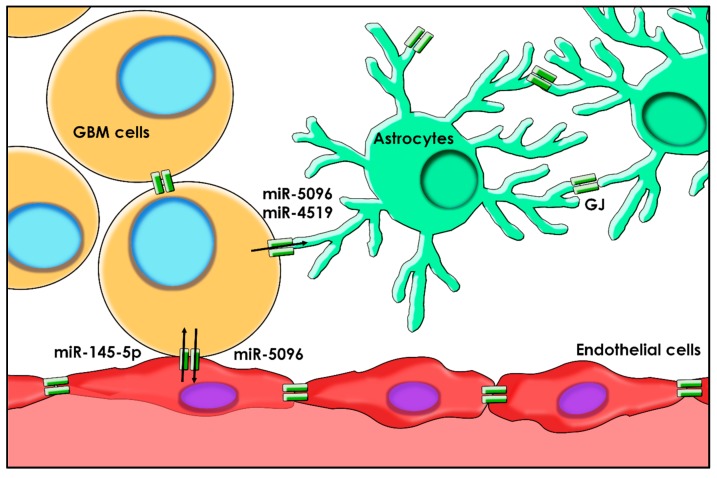
Glioblastoma cell—astrocyte—endothelial cell exchanging miRNAs via gap junctions (GJs): miR-5096 and miR-4519 are transferred from glioblastoma (GBM) cells to astrocytes and favors astrocyte recruitment to support tumor formation. The same miR-5096 is exchanged with miR-145-5p from the endothelial cell, promoting angiogenesis.

In the same study, the microRNA profile of astrocytes after co-culture with U87 cells revealed nine miRNAs to be increased: miR-4519, miR-5096, miR-3178, miR-3197, miR-4530, miR-4454, miR-4734, miR-3613-3p, and miR-20a-5p [57]. By GJ function blockade (via 18*α*-GA, Cx43-dominant negative mutant *T154A*, and noncontact co-culture), miR-4519 and miR-5096 were proved to be directly transferred through GJs from glioma cells to astrocytes (Figure 3). Conversely, the remaining seven miRNAs could either be transferred through exosomes or be transcriptionally upregulated.

With the aid of bioinformatics tools, Thuringer et al. identified the *KCNJ10* gene as a potential target of miR-5096 [69]. Since this gene encodes inwardly rectifying potassium channel Kir4.1, RT-PCR and Western blot analysis furtherly confirmed that miR-5096 mimic significantly decreases the levels of Kir4.1 in U87 and U251 cells. miR-5096 mimic was also shown to inhibit barium-sensitive current by this mechanism, and the authors suggested that the decrease in Kir4.1 may favor the assembly of cytoskeletal proteins (in particular, actin microfilaments) in filopodia projections, increasing glioma motility and invasion. Kir4.1 depletion by miR-5096 mimic, barium blockage, or small interfering RNA (siRNA) knockdown displayed a two-fold increase in the invasion rate of U87 and U251 cell lines. Moreover, miR-5096 also downregulates the expression of Cx43 in U87 cells, suggesting a pro-invasive effect. 

In the astrocytes-U87 co-culture, astrocytic inhibition of miR-5096 by anti-miR resulted in a decrease of GBM invasiveness. Since Kir4.1 is expressed exclusively in glial cells and miR-5096 is transferred from GBM cells to astrocytes, it is possible that this mechanism could also be responsible for the reactive astrogliosis that promotes glioma invasion. Research in this direction, along with miR-4519 bioinformatics predictions of possible targets, is needed in order to better understand GBM-astrocyte communication.

There are microRNAs that, although not proven to be transferred to astrocytes, were shown to be important to their neoplastic conversion. miR-10b, which is highly expressed in gliomas, as well as in brain metastases, was shown to induce astrocyte transformation. Additionally, miR-10b editing in oncogene-induced astrocytes decreased the number of transformed colonies [70]. Considering GBM’s ability to transform neighboring astrocytes into malignant cells, the transfer of miR-10b, as well as of other pro-oncogenic microRNAs, would be of great interest for future studies.

Since GJ transfer is bidirectional, it is a valid assumption that astrocytes could also transfer miRNAs to GBM cells. Currently, in vitro studies showed such transfer mechanisms only in brain metastases. Although astrocytes have been described to implement a defense mechanism against brain metastatic invasion through plasmin, there are numerous studies that show their protective effect on the neighboring tumoral cells, some of them by means of miRNA transfer [71]. 

Menachem et al. observed that, in a direct cell-cell contact co-culture of human lung tumor PC14 cells and mouse astrocytes, the transfer of miRNA from astrocytes via GJs increased tumor cells’ resistance to paclitaxel. mmu-miR-709, in combination with at least two miRs out of mmu-miR-16*, mmu-miR-1195, and endo-siRNA-1196, increased tumoral cells’ viability on an MTT assay. This suggests that miR-709 might play an important role in the tumor-protective effect mediated by the astrocytes [72]. Indeed, miR-709 has already been recognized as a pro-oncogenic microRNA, as its overexpression enhanced hepatocarcinoma proliferation, migration, and invasion through targeting glypican-5 (*GPC5*) [73]. However, the role of miR-709 in GBM is yet to be determined, especially in in vivo models.

Another miRNA, miR-19a, was proven to be transferred from astrocytes to metastatic cancer cells, leading to tumoral outgrowth. Zhang et al. showed that its transfer, mediated by exosomes, causes *PTEN* downregulation, which further leads to an increased secretion of cytokine chemokine ligand 2 (CCL2). In turn, CCL2 recruits Iba1+ myeloid cells that contribute to enhanced proliferation and reduced apoptosis [74]. Although miR-19a transfer from astrocytes to glioma cells, or vice versa, has not yet been detected, it is well-known that this particular miRNA is overexpressed in glioma tissues and is positively correlated with the tumor grade [75]. miR-19a has been found to target hundreds of genes in TargetScan and the Pictar database—some of which have been experimentally confirmed, such as the aforementioned *PTEN*, and which contribute significantly to glioma pathogenesis [76].

### 5.2. Oligodendrocytes—Glioblastoma Complicity?

Oligodendrocytes are the myelin-forming cells of the brain and have important neuron-protecting roles. Nonetheless, their function in glioma has not been as extensively studied as in the case of astrocytes. However, there is an established communication network between oligodendrocytes, astrocytes, and microglia that might be brought into play in the TME as well [77]. There are studies that indicate an anti-tumoral effect of oligodendrocytes, as tumor cells’ proliferation decreases in the presence of Wif-1 expressing oligodendrocytes [78]. Conversely, other studies showed a tumor-supportive role of oligodendrocytes [79,80]. Oligodendrocyte progenitor cells (OPCs) are the largest dividing population in the CNS and represent a cell type from which GBM may originate [81]. GBM cells were shown to migrate along the fasciculus axons, myelinated by oligodendrocytes, where numerous OPCs are found [80]. It is therefore reasonable to take into consideration a possible tumor-supportive role for OPCs as well.

The fact that most of GBM recurrences occur near the surgical resection in the macroscopically normal peritumoral parenchyma is a compelling argument for the changes induced by the tumor cells in this area. Indeed, in assessing the interaction between GBM and the glial cells, the tumor border region is of particular importance. By IHC of 19 GBM samples, Hide et al. compared cellular composition from three different regions—the tumor mass, the border area, and the peripheral area—showing that oligodendrocyte lineage cells (OLCs), including OPCs (Olig2+), together with macrophages/microglia (Iba1+ and CD163+), characteristically gather in the border areas; more specifically, in the invasion front [79,80]. 

In vitro, A172 and T98G cells were treated with human OPCs and macrophages condition medium. OPCs and macrophages were shown to promote stemness and the chemo-radio-resistance of GBM cell lines by secreting FGF1, EGF, HB-EGF, and IL-1β. Reciprocally, soluble factors derived from GBM cells were proven to increase OPCs’ viability [79]. This suggests that the recurrence and refractoriness to treatment after surgical resection are influenced by the presence of OPCs and macrophages in the border area.

In this “border niche”, as termed by the authors, five upregulated miRNAs (miR-219-5p, miR-219-2-3p, miR-338-3p, miR-27b, and miR-23b) and seven downregulated miRNAs (miR-630, miR-1246, miR-642b, miR-1181, miR-H18, miR-3195, and miR-3663-3p) were found throughout miRNA in situ hybridization of 89 GBM samples. The three miRNAs with the highest expression (miR-219-5p, miR-219-2-3p, and miR-338-3p) are all related to oligodendrocyte differentiation [79]. For instance, miR-219 and miR-338 have been previously identified as essential and sufficient to promote oligodendrocyte differentiation. This process takes place partly because they repress negative regulators of oligodendrocyte differentiation (*SOX6*, *HES5*, *FOXJ3*, *PDGFRA*, and *ZFP238*) [82,83]. miR-219 is not only important for the transition of OPCs to oligodendrocytes but also for the formation and maintenance of myelin [83]. 

Besides being involved in cellular differentiation, miR-219 and miR-338 also have a suppressing effect on astrocyte activation, as they reduce the expression of Vimentin and Serpina3. In addition to the aforementioned actions, miR-219 and miR-338 have tumor-suppressing effects. For instance, the highest expressed miRNA at the border region, miR-219-5p, has been previously reported to act as a tumor suppressor in GBM, as well as in other types of tumors [79]. Particularly, miR-219-5p represses *EGFR* expression and, thus, contributes to the increased activity of the RTK pathway. It therefore inhibits the proliferation, anchorage independent growth, and migration of glioma cells. In addition, miR-219-5p inhibited MAPK and PI3K pathways in glioma cell lines [84]. 

There seems to be well-founded evidence pointing to the participation of miR-219 and miR-338 in the GBM microenvironment, but their exact role in the communication network developed by the multiple type of cells involved is yet to be determined. Exosomes from oligodendrocytes were shown to be transferred to microglia by macropinocytosis, and the pro-inflammatory signals inhibit their internalization in vitro [85]. Studying the miRNAome of oligodendrocytes-derived exosomes will definitely open a new area of research.

## 6. Thirst for Blood—Angiogenesis in GBM

GBM expansion is coupled with the formation of new pathological blood vessels, being the most vascularized brain tumor in humans [86]. Endothelial proliferation from this tumor is a direct measure of its malignancy, and one of the most dreaded complications is the occurrence of vasogenic brain edema through blood-brain-barrier (BBB) leakage. 

### 6.1. Intercellular Communication—Enemy Propaganda

The communication between GBM cells and the surrounding microenvironment stands as a testament for their complexity and adaptability. Numerous processes are responsible for GBM expansion, and angiogenesis, cellular communication being at the forefront. 

Increased levels of the long non-coding RNA (lncRNA) H19 have been observed in numerous cancers, also being involved in the invasion, angiogenesis, stemness, and tumorigenesis of GBM cells [87,88]. It is involved in the cell-cell communication between GSCs and ECs and upregulates the expression of the angiogenic factor VASH2 by repressing miR-29a. Another lncRNA, XIST, possibly enables GBM angiogenesis by rising the promoter activity of chemokine receptor 7b (CXCR7) or capturing and inhibiting miR-429 in the manner of a molecular sponge [89,90]. 

As previously mentioned, GBMs cells express Cx43 and construct GJIC [45]. Using a 3D migration model, Aftab et al. demonstrated that a decline in GJIC stimulates glioma cell migration [66]. In their in vitro study, Krcek et al. have shown that VEGF incubation and radiation therapy significantly increase GJIC in U251 cells [45]. In astrocytes, VEGF has been shown to significantly enhance GJIC, positively affecting cellular proliferation and motility, a feature most likely inherited by GBM cells as well [91]. 

GJs also play a role in the transfer of functional miRNAs between GBM cells and human microvascular endothelial cells (HMECs) [46]. Both miR-145-5p, which is expressed in HMECs and virtually absent in U87 cells, and miR-5096, which is mainly present in U87 and scantily detected in HMECs, have been demonstrated to pass from one cell type to the other via GJs in a time-dependent manner in vitro (Figure 3). Moreover, miR-5096 promotes GBM invasiveness and possibly stimulates angiogenesis after being transferred into ECs, whereas miR-145-5p acts as a tumor suppressor and is downregulated in the early stages of GBM development [92]. Since carbenoxolone inhibited the transfer of both miRNAs, it is highly likely that they use the same GJIC pathway to accomplish their transfer. Moreover, GJIC between GBM cells and HMECs lose their functionality in vitro after a few days; however, miR-5096 still manages to pass in a less efficient manner, probably through exosome release [69,93,94]. 

A recent research revealed that GSCs-derived exosomes were fully equipped for the stimulation of angiogenesis via their intrinsic miR-21, which triggers the angiogenic capacity of ECs [58]. In GSCs, miR-21 mimics lead to higher mRNA levels of VEGF. Fluorescent labeled exosomes derived from GSCs were proven to be integrated into ECs when co-cultured. Furthermore, miR-21 was highly increased in ECs after the transfer, and higher levels of VEGF were measured. Consequently, enhanced tube formation and migration abilities of ECs were detected [58]. Considering that GBM cells were also proven to release miR-21-enriched exosomes, this mechanism could be extended in the GBM cell-EC communication. Notably, angiogenesis was also stimulated in GBM via exosomes supplemented with the lncRNA POU3F3 [95]. 

TNTs have been recently proven to be involved in cell-cell communication between distant cells. Apparently, pericyte-derived TNTs energetically explore the neighboring microenvironment, seeking and connecting with targeted vessels [96]. This would likely imply that TNTs possess a primordial role in both physiological and pathological angiogenesis in the brain. The implications of TNTs in GBM and their role in tumor aggressiveness and angiogenesis remain to be studied.

### 6.2. AngiomiRs—Pulling The Strings?

Lately, a group of miRNAs, labelled angiomiRs, have been acknowledged as essential contributors to GBM neo-vascularization, performing on either malignant cells or adjacent tumor-associated cells [97,98]. 

miR-296 is such an angiomiR, determined to be upregulated in ECs in the company of GBM cells (U87) or angiogenic growth factors like VEGF and correlated with increased endothelial cell tube formation and amplified tumor vascularization [99,100]. Among the computationally predicted target of miR-296 stands hepatocyte growth factor-regulated tyrosine kinase substrate (*HGS*), which was furtherly confirmed by luciferase assay. HGS is a potent regulator of growth factor receptors, including VEGFR2, and through this mechanism, the ECs adapt to angiogenic stimuli.

As Yue et al. have stated, miR-205 acts as a GBM suppressor by targeting *VEGFA* and is significantly underexpressed in glioma cell lines (U87 and LN229) and tissue samples [101]. Similarly, miR-29b has been linked to attenuating GBM angiogenesis and stemness by directly inhibiting B-cell lymphoma 2 homolog, Bcl2-like 2 (*BCL2L2*; also referred to as Bcl-w), which is amply expressed in the mesenchymal subtype of GBM [102]. Human umbilical vein endothelial cells (HUVECs), as well as HBMECs, expressed lower levels of angiopoietin-2 (Ang-2) and VEGF after exposure to miR-29b mimic. 

Another microRNA, miR-124-3p, was shown to be underexpressed in five human glioma tissues [103]. Luciferase assay indicated miR-124-3p specifically targeted *NRP1* and controlled GBM proliferation, growth, and angiogenesis via the PI3K/Akt/NF-κB and KRAS/ERK signaling pathways. The supernatant of miR-124-3p transfected U87 and U251 cell cultures restricted tube formation of HUVECs. In a previous study, Zhang and collaborators found that the NRP-1/GIPC1 signaling pathway possesses a key role in GBM development, hinting that miR-124-3p may play an important part in the inhibition of GBM progression [104]. However, a larger sample is needed to provide more statistical power regarding the specificity of miR-124-3p deregulation in GBM.

miR-129-5p is downregulated in GBM clinical samples and also in cell lines (including U87, U251, T98G, and A172) compared to normal human astrocytes [105]. Its overexpression inhibits GBM cell proliferation and angiogenesis by targeting the noncanonical Wnt molecule Wnt5a (confirmed with luciferase assay). Cultured in the supernatant of miR-129-5p overexpressing GBM cells, HBMVECs diminished their angiogenic ability and tube formation. Silencing of Wnt5a leads to a blockage in the protein kinase C (PKC)/ERK/NF-κB and JNK pathways, which Zeng et al. have also proven to have an inhibitory effect on GBM growth in vivo.

There is surmounting evidence regarding the relationship between hypoxia and angiogenesis in GBM in the sense that hypoxia persists regardless of the increased tumor vasculature [106]. According to Agrawal et al., a hypoxic GBM microenvironment upregulates miR-210-3p, miR-1275, miR-376c-3p, miR-23b-3p, miR-193a-3p, and miR-145-5p, whereas miR-92b-3p, miR-20a-5p, miR-10b-5p, miR-181a-2-3p, and miR-185-5p appear to be downregulated in U87 and U251 cell lines [107]. Intriguingly, several hypoxia-induced miRNAs have been shown as overexpressed in malignant gliomas, advocating that hypoxia could represent one of the principal factors that shape the miRNA designation of GBM. In this study, miR-210-3p has been especially linked to GBM hypoxia/stress/chemo-resistance promotion, thus appointed as an oncomiR in GBM. In a study ascertaining hypoxia-mediated miRNA levels in GBM before and after bevacizumab therapy, high amounts of miR-10b and miR-21 were observed in the serum of most of the individuals during the bevacizumab treatment period when compared to pretreatment levels [108]. The researchers concluded that miR-10b and miR-21 may expose the antiangiogenic consequence of bevacizumab therapy, but their implications as tumor-response biomarkers is as of yet unclear. In another study, it was highlighted that miR-21-silencing resulted in an increased sensitivity to the antiangiogenic drug sunitinib in GBM [109].

As shown by Fang and collaborators, miR-93 serves as an oncogene by augmenting GBM cell survival, tumor growth, and vascular development [110]. It accomplishes these feats, at least partially, by decreasing integrin-β8 expression. Thus, U87 cells were transfected with miR-93 and injected subcutaneously into mice. Tumor sections were analyzed by IHC showing increased levels of blood vessel marker CD34, lower integrin-β8, along with high densities of vessels. Ypen (rat ECs) were cultured in spent medium from miR-93-transfected U87 cells, and the ECs showed increased proliferation. Knockout of integrin-β8 genes in embryonic neuroepithelial cells lead to endothelial cell hyperproliferation due to the defective activation of latent TGF-β [111]. 

By directly inhibiting miR-299, the lncRNA taurine upregulated 1 (TUG1) heightens tumor-induced angiogenesis [112]. Cai et al. also revealed that there is a mutual suppression between TUG1 and miR-299 within the same RISC. In vivo, U87 and U251 xenograft models also showed ablation of TUG1 diminished the tumor microvessel density. 

Similarly, miR-128 is also able to regulate angiogenesis by repressing P70S6K1, a kinase located upstream of HIF-1α and VEGF [113]. Restoration of normal miR-128 levels may lessen angiogenesis and invasiveness of U87 and U251 cells, whereas a sustained expression of P70S6K1 can moderately salvage the inhibitory role miR-128 plays on cancer growth. In vivo, a subcutaneous mouse model of miR-128 overexpressing U87 cells displayed high levels of CD31 (vessel markers), suggesting elevated angiogenesis.

It was confirmed that GBM cells deprived of nutrients or exposed to chemotherapy agents displayed increased levels of miR-17 [114]. By transfecting U87 and U343 cells with miR-17, the researchers managed to extend malignant cell survival under the aforementioned inhospitable conditions. miR-17 also increases stimulated cell motility, aggressiveness, and the formation of nanotubes—phenotypes which were the effect of miR-17 targeting *PTEN*. As an aftermath of PTEN reduction, HIF1α and VEGF were overexpressed and prompted a cascade of angiogenesis and migration. Additionally, the ectopic expression of miR-17 was noticed to expedite the proliferation of GSCs, which in turn exhibited an augmented ability to establish colonies and neurospheres and presented greater CD133 levels.

In vitro, reduction of miR-10b resulted in a decrease of the invasiveness, angiogenesis, and expansion of the mesenchymal subtype GBM, while significantly extending survival in mice models [115]. The authors proved that the pleiotropic behavior of miRNA-10b was owing to its inhibition of several tumor suppressors, counting *CYLD*, *FOXO3*, *HOXD10*, *NOTCH1*, *PAX6*, *PTCH1*, and *TP53*. 

Other notable angiomiRs include miR-7, miR-15b, miR-16, miR-31, mir-124, miR-125b, miR-128, miR-143, and miR-331-3p and are commonly decreased in GBMs [97,100,116]. miR-7-5p acts as a tumor suppressor that reduces GBM microvascular endothelial cell proliferation, possibly by suppressing the RAF1 oncogene [114]. miR-125b downregulation instigates heightened expression of its target, Myc-associated zinc finger protein (MAZ), a transcription factor that controls VEGF levels. As such, a diminished expression of miR-125b in malignant cells stimulates neo-vascularization of tumors [117]. 

## 7. Glioma Stem-Like Cells

Cancers of all types are now known to consist of highly diverse cell populations with distinct genetic modifications and varied levels of differentiation [118]. Therefore, in a caricatural way, tumors resemble the heterogenic architecture of healthy tissues, including niches with histological and functional particularities. However, while in normal tissues there is a very rigid cellular hierarchy concerning the distribution of stem and non-stem cells, tumors show increased stem-cell plasticity, with cells being able to transition in and out of the stem-like phenotype in order to adapt to a changing environment [119]. In order to do so, normal stem cells communicate with their progenitors through mRNA and miRNA released in EVs, thus maintaining normal cell populations and hierarchy [120]. Consequently, the role of EVs containing microRNAs must also be considered in the intratumoral cell hierarchy [121]. 

Given the important role of EVs in determining cell hierarchy and regulating the microenvironment, it is probable that the same mechanisms also exist in GBM and may be involved in the therapeutic response [122]. GBM is a highly heterogenic tumor, with GSCs being responsible for tumor progression, drug resistance, and relapse. Compared to normal stem cells, GSCs have a different transcriptional, epigenetic, and metabolic profile, even if the surface markers and self-renewal capacity are similar [123]. Epigenetic regulation allows the generation of different transcriptional profiles, thus maintaining the GSCs phenotype, while also integrating signals from the TME [124]. Currently, there is no agreement regarding the definition of GSCs. Gimple et al. employ a series of functional criteria, including tumor-initiating capacity following serial transplantation, self-renewal, and the ability to recapitulate tumor heterogeneity [125]. 

RNA-binding proteins (RBPs) are regulators of post-transcriptional events. In GBM, high concentrations of RBPs are associated with poor prognosis [126]. Noncoding RNAs, including lncRNAs and miRNAs, also play a role in gene expression, targeting mRNAs at the posttranscriptional level. In GBM, stem-associated microRNAs have been identified to be involved in tumor progressions and radio-resistance, many of which are correlated with a poor prognosis [127]. 

Lang et al. found miR-10a and miR-10b to be upregulated, whereas miR-124-3p and miR-874 were downregulated in GSCs [128]. Additionally, an miRNA signature associated with stem-like phenotype in GBM was identified by Sana et al.: highly expressed miR-9-3p, miR-93-3p, miR-93-5p, miR-106b-5p, miR-124-3p, miR-153-3p, miR-301a-3p, miR-345-5p, and miR-652-3p. miRNAs expression was negatively correlated with the survival, independent of *IDH1* mutation status [129]. However, miR-124-3p showed controversial expressions in these studies. Although it was demonstrated to be downregulated in GBM tissues and to act as a tumor suppressor, further research is needed to determine its role in GSCs. Meta-analyses of future studies will probably reveal accurate signatures of GSCs. 

Figueroa et al. showed that glioma-associated mesenchymal stem cells (GA-hMSCs), a newly identified stromal component of GBM, release exosomes containing miRNAs that are then internalized by GSCs, resulting in increased GSC tumorigenicity. For instance, miR-1587 and miR-3620-5p were shown to increase the proliferation, and miR-1587 also inhibited the hormone receptor co-repressor-1 (*NCOR1*), a tumor suppressor of GSCs, after exosomal transfer [59]. GSCs pre-treated with GA-hMSC-derived exosomes were intracranially injected in mice and showed lower survival and increased tumor volumes. 

## 8. The Immune System

### 8.1. The Innate Immune Cells—TAMs

Chronic inflammation is already established as a preneoplastic lesion for many types of cancer. Nevertheless, anti-inflammatory mediators were also identified to promote malignancy. As key regulators of these processes, myeloid-derived and tissue-resident macrophages have gained considerable recognition as crucial participants in the TME. 

Tumor-associated macrophages/microglia (TAMs) are a major constituent of GBM, representing 30%–50% of its cells [130]. In GBM, a bidirectional crosstalk between tumor cells and TAMs is set, resulting in particular phenotypes of the immune cells, as well as specific functional programs. This polarization of macrophages can be simplified throughout a binary scheme derived from in vitro exposure to signals: classical activation (M1 polarization) and alternative activation (M2 polarization) [131]. The classical activation (by lipopolysaccharide, TNF-α, and IFN-γ) leads to a pro-inflammatory state, secreting IL-1β, IL-6, IL-12, and TNF-α and expressing CD68, CD80, and CD86 on their surface. The alternative activation (by IL-4, IL-10, and IL-13) leads to an anti-inflammatory phenotype, producing IL-10 and TGF-β and is characterized by specific membrane markers such as CD163, CD206, and Arg1 [131]. Intracellularly, M1 macrophages are distinguished from their counterparts through the STAT1 pathway, which is activated, while M2 macrophages feature an activated STAT3 signature [132]. Some authors suggested that TAMs are M1 polarized in the initiation stage of cancer, but they later acquire a M2 polarization as the tumor progresses [133]. The TAMs resemble the M2 phenotype and function in other solid tumors and predict a poor outcome of the patients [134]. However, brain trauma animal models revealed not only a mixture of M1 and M2 populations but also the co-expression of canonical markers in single cells [135]. Accordingly, the M1/M2 polarization model is not suitable for in vivo characterization of macrophages. 

Furthermore, microglia need to be distinguished from myeloid-derived macrophages, since they have different ontogeny and gene expression. The M1/M2 polarization of microglia is also not applicable in vivo mainly because the definition of polarization derives from in vitro exposure to stimuli, and most of them are not present in the CNS [136]. Secondly, isolated ex vivo and in vitro microglia show numerous differences, from epigenetic profiles to functions. Particularly, in GBM, the heterogeneity in space and time favors adoption of different states, even in the same cell. For these reasons, M1 and M2 patterns are insufficient for a complex description. In reality, the plethora of signals can activate intermediate programs, leading to the secretion of both pro- and anti-inflammatory cytokines. The pro-inflammatory cytokines regulate diverse malignancy features, whereas the anti-inflammatory microenvironment, besides pro-tumorigenic roles, also promotes immune evasion. Thus, glioma-associated microglia cannot be described as pro-inflammatory or anti-inflammatory but as tumor-supportive. 

Therefore, the M1/M2 polarization of microglia/macrophages must be critically perceived from the perspective of cell culture-based in vitro models, studies on more complex models such as organotypic slice culture or GBM organoids being absolutely necessary in the future for a more accurate characterization of their activation.

#### 8.1.1. Microglia Recruitment—The First Response

The first immune cells to react towards GBM are the resident immune cells of the CNS: microglia. In contrast with monocytes/macrophages that originate in the hematopoietic stem cells from the bone marrow, microglia have a controversial origin linked with the early embryonic development. Therefore, as opposed to their myeloid-derived counterparts, microglia consist of a stable population with low capacity of self-renewal [130]. In healthy individuals, microglia are located in the entire CNS in a “surveillant” state (ramified microglia), previously termed as “resting microglia”, which act as a housekeeper in removing metabolic products, damaged cells, as well as monitoring discrete changes in the microenvironment [137]. As a response to immunological stimuli, microglia change their phenotype, adopting a fully active form. 

As specified before, Cx43 is overexpressed in reactive astrocytes and forms a developed network of gap junctions and hemichannels. By eliminating astrocytic Cx43 in a mouse model, Sin et al. observed that the reactive microgliosis is reduced at the tumor border compared to the wild-type Cx43 animals [56]. Thus, astrocytic Cx43 is essential for microglia recruitment. However, the signaling molecule that mediate this interaction has not been studied yet. A pertinent hypothesis is that TNF-α and IL-1β from M1-like microglia could disrupt GJs in astrocytes and activate the hemichannels via p38-MAPK [138]. Through the hemichannels, astrocytes release ATP that activates microglia and promotes their migration [139]. Although microglia were proven not to form functional GJs in vivo, miRNAs transferring from reactive astrocytes to microglia by hemichannels is plausible and should be investigated [140].

Glioblastoma-derived EVs carries high levels of miR-21 and miR-451 that are taken up by microglia [60,62]. As previously mentioned, miR-21 is one of the most crucial oncomiRs in GBM cells, promoting invasion and cell survival. Instead, miR-451 seems to promote proliferation of GBM cells in normoglycemic conditions. Overall, through the suppression of their target mRNAs, these miRNAs favor the migration and proliferation of microglia.

By intracranial injection of mouse GL261-derived EVs in a miR-21-null mouse model, Abels et al. illustrated significantly high levels of miR-21 in microglia and macrophages [62]. To differentiate surveillant microglia from activated macrophages, anti-CD11b and anti-CD45 antibodies were used. Consequently, miR-21 from EVs was shown to be enhanced in microglia compared to macrophages. The mRNA transcriptome of microglia revealed down-regulation of *NFAT5*, *BMPR2*, *BTG2*, *RHOB*, and *KBTBD2*. In vitro, microglia isolated from miR-21-null mice were transfected with a miR-21 mimic, and qRT-PCR confirmed similar expression patterns of the target genes [62]. Although these genes affect different behaviors, the downregulation of *BTG2* was the most significant and increased the microglial proliferation (Ki67 overexpression). 

Another line of evidence was provided by Van der Vos et al., who detected high levels of miR-21 and miR-451 in EVs derived from two patient-derived GBM cell lines (GBM 11/5 and GBM 20/3). Diverse microglial cells (murine KW3, primary mouse microglia, and human adult microglia) were exposed in vitro to these GBM-EVs, and consequently, both miR-21 and miR-451 were significantly increased. qRT-PCR and Western blot analysis depicted decreased expression of the target genes *PTEN* (for miR-21) and *MIF* (for miR-451). Moreover, both miR-21 and miR-451 showed a synergic effect in downregulating c-Myc. Since c-Myc is often overexpressed in malignant tumors, the inactivation in microglia is counterintuitive and is supposed to promote shifts in gene expression programs. In glioma cells, miR-451 was shown to repress the CAB39/LKB1/AMPK pathway [22]. Although the downregulation of CAB39 in microglia was certified in vitro after miR-451 uptake (by qRT-PCR but not by Western blot), this pathway was not fully investigated. It is possible that this mechanism promotes the proliferation of microglia via mTOR. 

**Figure 4:**
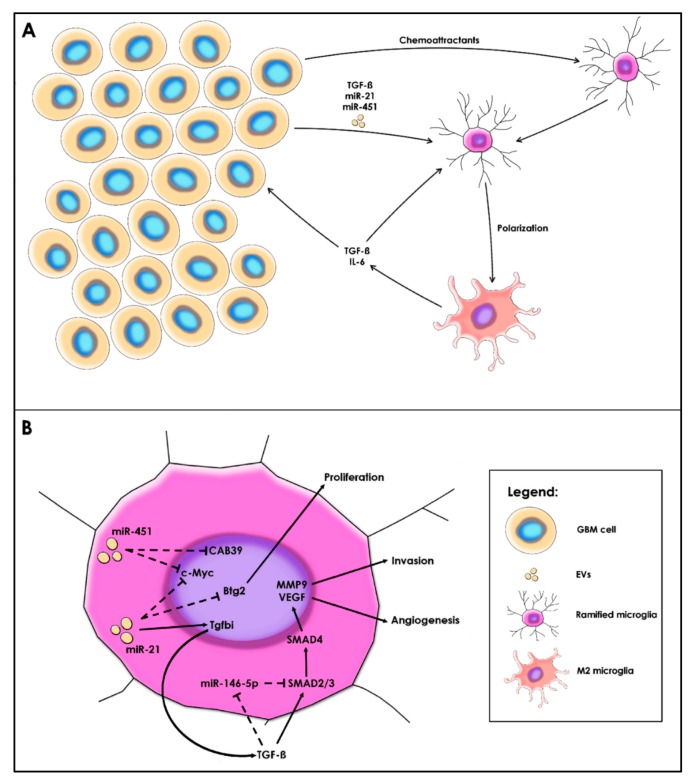
(**A**) Microglia recruitment and M2 polarization under the influence of soluble factors and miRNAs from GBM cells. (**B**) miR-21, miR-451, and miR-146-5p roles in the activation of microglia (arrows—activation; dotted T-bars—inhibitory effect).

Interestingly, the previous studies had contradictory results regarding the TGF-β levels in microglia. Abels et al. showed that *FASL* and *TGFBI* mRNA were significantly higher in microglia after the uptake of miR-21, while Van der Vos et al. suggested the high levels of TGF-β are due to direct uptake from EVs and not from microglial synthesis induced by miR-21 [60,62]. Since different GBM cell lines were used in these studies, it is normal for the composition of EVs to vary, making it a possible explanation for this discrepancy. However, both studies agree that microglia possess high levels of TGF-β, suggesting the M2-like polarization. Forming a positive-feedback loop, TGF-β from GBM cells and microglia acts as a chemoattractant and promotes microglial migration towards the GBM microenvironment. Moreover, TGF-β activates the SMAD2/3/4 complex in microglia and overexpresses VEGF and MMP9, thus supporting angiogenesis and, respectively, migration of both microglia and GBM cell. M1-activated microglia normally express high levels of miR-146-5p, which directly inhibit SMAD4 expression. However, in glioma-associated microglia, miR-146-5p is significantly decreased [141]. This suggests that, in vitro, M1 microglia switch to the M2 state and assure the maintenance of the M2 subpopulation through paracrine secretion (Figure 4). 

Another mechanism of M2 polarization was observed by Li et al. [142]. In GBM samples, tumor-suppressive miR-627 is significantly downregulated, while lncSNHG15 and CDK6 are overexpressed [142,143]. Using temozolomide-resistant (TMZ-R) and temozolomide-sensitive (TMZ-S) cells from patient samples, Li et al. showed that TMZ-R cells display higher levels of lncSNHG15 in comparison to TMZ-S cells, along with stemness features such as higher expressions of Sox2, β-catenin, CD133, and EGFR. Since lncSNHG15 silencing yielded TMZ sensitivity, this supports the association of lncSNHG15 with TMZ resistance. To study GBM-microglia interaction, HMC3 microglia were co-cultured with TMZ-R, respectively with TMZ-S. Both GBM cell lines promoted the M2 polarization of microglia, increasing mRNA levels of the CD206, CD163 markers, while not influencing M1-cytokines (TNF-α and IFN-γ) expression. ELISA assays confirmed higher levels of M2-cytokines (IL-6 and TGF-β) but not of M1-cytokines. Furthermore, TMZ-R cells were shown to promote the M2 polarization of microglia more effectively than TMZ-S. Blockade of CDK6 in TMZ-R cells by palbociclib resulted in a decrease in lncSNHG15 expression, suggesting that CDK6 is an important upstream signal for lncSNHG15 [142]. Instead, the role of miR-627 is to suppress CDK6 expression, leading also to lncSNHG15 downregulation. In a TMZ-R patient-derived xenograft mouse model, the combination of TMZ and palbociclib delayed tumor growth, and tissue analysis by RT-PCR showed the lowest level of β-catenin, Sox2, CDK6, and lncSNHG15. Immunohistochemistry was used to determine CD206 in tumor samples showing the lowest expression. However, as polarization is inappropriate for in vivo characterization of microglia, M1 markers were not searched, and CD206 cannot distinguish microglia from macrophages; these results should have been investigated using a larger set of IHC markers. Taking together, at least in vitro, resistant clones of GBM promote M2 polarization in a miR-627/CDK6/lncSNHG15-dependent manner.

Overall, GBM cells, GSCs, and reactive astrocytes promote the proliferation and migration of microglia, leading to microgliosis in the tumor border. As mentioned before, oligodendrocyte progenitors and macrophages/microglia cooperate to induce stemness features and chemo-resistance in the border niche [79,80]. miR-219-5p, miR-219-2-3p, and miR-338-3p are the main overexpressed miRNA in this area and play roles in oligodendrocyte differentiation. However, miR-219-5, which is the highest expressed, also plays anti-apoptotic roles in macrophages [144]. In vivo, miR-219 was shown to downregulate the pro-inflammatory cytokine TNF-α but not IL-1 in microglia [145,146]. Still, in this study, markers of M2 polarization were not investigated. The extensive roles of miR-219-5p, miR-219-2-3p, and miR-338-3p remain to be elucidated in TAMs.

#### 8.1.2. Monocytes—An Endless Reservoir of TAMs

Although microglia are the main immune cells in the brain, GBM promotes BBB disruptions and favors the entrance of peripheral immune cells. Monocytes infiltrate into GBM from the periphery and further differentiate into macrophages. It was demonstrated in a chimeric GL261 mouse model that more than 25% of TAMs are myeloid-derived after three weeks, suggesting that the GBM composition in TAMs evolves from a steady microglial population to a combination between microglia, monocytes, and macrophages [147]. 

Circulating monocytes are normally cytotoxic to malignant cells. Nonetheless, once they have entered the tumor tissue, direct cell-cell contact between glioma cells and monocytes change their behavior. GBM cells express adhesion molecules such as vascular cell adhesion molecule 1 (VCAM-1, CD106) and intercellular adhesion molecule 1 (ICAM-1, CD54) that interact with α4β1 integrin from the monocyte membrane. Liu et al. identified by IHC a positive correlation between VCAM-1 expression and the glioma grade [148]. In addition, treatment of U251 and ALTS1C1 cells with TNF-α increased VCAM-1 expression (Western blot and PCR), and flow cytometry confirmed the localization of VCAM-1 on the surface membrane of the GBM cells. Fluorescent-labeled THP-1 monocytes were co-cultured with the TNF-α-treated U251 and ALTS1C1 cells, showing enhanced binding activity. siRNAs were used to inhibit VCAM-1 synthesis and decrease monocyte adhesion. On the other hand, anti-integrin α4 and β1 antibodies influenced adhesion in the same manner. By formation of the VCAM-1-α4β1 integrin complex, recruited adherent monocytes produced high levels of TNF-α and IFN-γ but not of Arg1, IL-10, and CD163. Intriguingly, these markers suggest a M1 polarization of monocytes. miRNA microarray profiling showed that TNF-α downregulates the expression of miR-181a and miR-181b in GBM cells. Furthermore, transfection of U251 cells with miR-181b or miR-181a mimics repressed VCAM-1 expression. These miRNAs normally suppress the activity of protein phosphatase 2A (PP2A), shown to regulate important oncogenic drivers. Thus, by this mechanism, adherent monocytes favor the phosphorylation of STAT3, p38, and p65 and induce the overexpression of VCAM-1, facilitating a positive feedback-loop in the recruitment of monocytes. 

**Figure 5:**
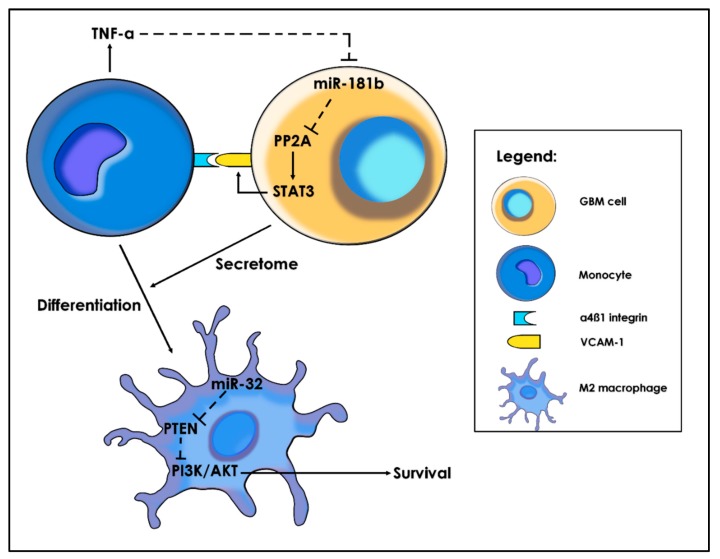
Vascular cell adhesion molecule 1 (VCAM-1)-mediated monocyte adhesion and differentiation towards the M2-macrophage (arrows—activation; dotted T-bars—inhibitory effect).

Nonetheless, exposed to the GBM secretome, monocytes differentiate into M2-macrophages, suggesting a similar switch from the pro-inflammatory state to the anti-inflammatory one, as seen in microglia. Bao et al. demonstrated that cultivating human monocytic cell line THP-1 with U87 supernatant resulted in significantly increased miR-32 in monocytes [149]. miR-32 inhibits PTEN expression, consequently activating the PI3K/AKT signaling pathway (Figure 5). In turn, activated PI3K/AKT inhibits apoptosis by downregulating Bad and active cas-9 and upregulating Bcl2. Furthermore, miR-32 mimics in monocytes promoted the polarization towards the M2 macrophage phenotype. As a result, transformed macrophages expressed M2 markers (CD206, CD204 and CD163) and secreted chemo-attractants (CCL18, CCL22 and CXCL2) and pro-tumorigenic chemokines (IL-10, VEGF and TGF-β), leading to an increase in GBM proliferation and migration.

Thus, these two independent studies showed conflicting results regarding the differentiation and polarization of monocytes. However, the polarization of monocytes is still debatable, and other studies depicted a mixture of M1 and M2 markers in recruited monocytes in vitro [150]. We recommend research in this area using fluorescent-tagged monocytes in GBM animal models (GFP bone marrow chimeras) and IHC analysis of both M1 and M2 markers in myeloid-derived cells. Moreover, immediately isolated ex vivo monocytes could offer a more detailed perspective regarding miRNAs and the expression of the M1/M2 cytokines.

#### 8.1.3. Macrophages—The Biggest Enemy 

The major miRNA involved in M2-macrophage maintenance is also miR-21. As we have previously mentioned, GBM cells release miR-21-containing EVs and, similarly to microglia, both monocytes and macrophages take up miR-21 [62]. However, this process is not as significant as seen in microglia, probably because macrophages have phagocytic activity and rather ”digest” than englobe these EVs. In macrophages, miR-21 downregulates STAT1. Since the JAK2/STAT1 pathway mediates the IFN-γ-signaling required for M1 polarization, miR-21 promotes a shift to the STAT3 pathway, which in turn amplifies the intracellular level of miR-21 [151]. STAT3 activates important downstream targets in macrophages, including CD163 and IL-10, both markers of M2 polarization.

Moreover, macrophages also release EVs enriched in miR-21 that are transferred to glioma cells [61]. Chuang et al. illustrated that clinically isolated TAMs from GBM patients released exosomes in the culture medium. The origin of exosomes was confirmed, as they expressed CD9, CD63, and CD81. In addition, U87 cells were treated with miR-21 mimic, and an increase in Sox2, STAT3, Wnt, and Nestin was observed. 

All-in-all, through this bidirectional miR-21 exchange, STAT3 activity increases in both GBM cells and macrophages, promoting invasion, proliferation, angiogenesis, and resistance to treatment. In vivo, administration of STAT3 inhibitor pacritinib reduced tumor size in a mouse model bearing LN18 cells co-cultured with TAMs exosomes. 

Another feedback-loop of M2-macrophage maintenance is mediated by miR-340-5p [152]. This miRNA has been reported to be downregulated in GBM and is inversely correlated with tumor size and recurrence rate. The low expression of miR-340-5p significantly upregulates POSTN and latent TGF-beta-binding protein 1 (LTBP-1) in GBM cells. In turn, POSTN mediates αVβ3 integrin signaling and increases TAMs’ recruitment. Moreover, LTBP-1 increases the M2 polarization, since this protein enhances TGF-β activity [153]. The recruited M2-TAMs inhibit miR-340-5p expression in GBM cells through the TGF-β/High mobility group AT-hook 2 (HMGA2) pathway, assuring a continuous feedback loop and maintaining GBM tumorigenicity. 

**Figure 6:**
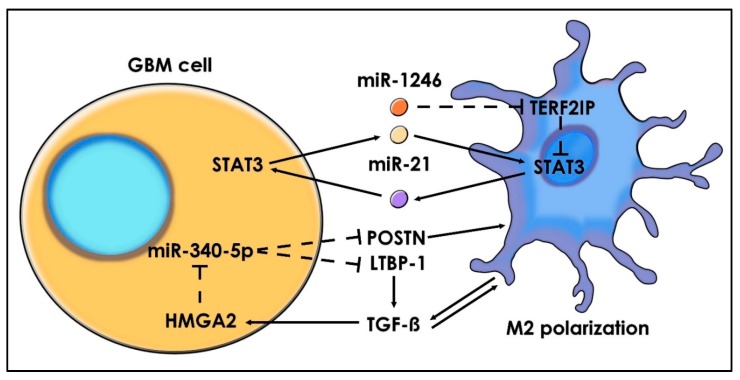
M2-macrophage maintenance by miRNA dysregulations in GBM. Hypoxic-derived exosomes contain miR-1246, which modulate STAT3 activity, alongside miR-21 EVs released from both GBM cells and M2-macrophages in order to support each other by STAT3 activation. miR-340-5p downregulation is a key mechanism of communication, since its downstream targets contribute both to M2 polarization and the maintenance of miR-340-5p underexpression; (arrows—activation; dotted T-bars inhibitory effect).

In the hypoxic niche, glioma-derived exosomes have different miRNA signatures. Thus, miR-1246 was found as the most abundant miRNA in hypoxic exosomes [154]. miR-1246 is internalized by macrophages and exerts its effect by direct silencing of telomeric repeat-binding factor 2-interacting protein (*TERF2IP*). Consequently, the downregulation of *TERF2IP* activates the STAT3 pathway and inhibits NF-κB signaling, facilitating the macrophage to assume the M2-like polarization in the hypoxic niche (Figure 6).

### 8.2. Adaptive Immune Cells

Monocytes are not the only cells entering the brain parenchyma, lymphocytes being able to act in the same manner. Natural killer T lymphocytes (NKT) are a small subpopulation of T cells involved in the release of pro-inflammatory cytokines (IFN-γ, TNF-α, IL-2, IL-4, IL-13, IL-17, and IL-21) [155]. Generally, NKTs have anti-cancer responses, but as the tumor progresses, NKT cells promote immune evasion. 

**Figure 7:**
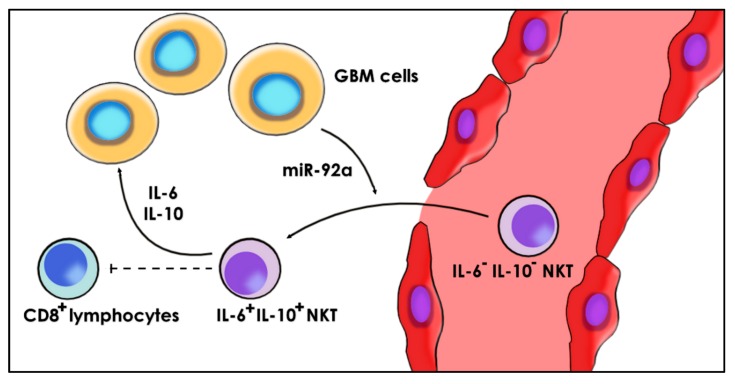
Natural killer T cells (NKT) differentiation and roles of the immunotolerant NKT in glioblastoma maintenance and immune evasion (arrows—activation; dotted T-bars—inhibitory effect).

Tang el al. observed that, in glioma patients, while only 5.8% of peripheral NKT expressed IL-6 and IL-10, 92.4% of cells in glioma tissues were IL-6+ IL-10+. Moreover, co-cultivation of NKT cells with GBM cells induces IL-6+ IL-10+ NKT cells. While normal NKTs have anti-tumor effects, IL-6+ IL-10+ NKT cells lose these functions. Co-cultured NKTs produce lower levels of perforin, FasL, and IFN-γ. In addition, they markedly reduce glioma cells apoptosis and suppress the anti-CD3/CD28-induced CD8+ T cells proliferation [156]. These results indicate that NKT cells differentiate in the tumor microenvironment in order to support the glioblastoma through IL-6 and IL-10. The key contributor to this differentiation is glioma-derived miR-92a. The frequency of IL-6+ IL-10+ NKT is significantly increased by adding miR-92a in the culture and is abolished by the antisense of miR-92a. As previously reported, miR-92a is overexpressed in GBM and exerts anti-apoptotic effects [157]. Activation of glioma cells by TRL4 agonists majorly increases the expression of miR-92a, suggesting that the pro-inflammatory state leads to immunotolerant NKT cells (Figure 2). It is currently not known whether miR-92a is transferred to NKT via EVs or acts through the modulation of the GBM secretome. However, GL26 were cell-derived exosomes shown to decrease the number and activity of CD8+ lymphocytes, as well as the levels of IFN-γ and granzyme B that are normally produced by CD8+ lymphocytes and NKT cells [158]. Further research is needed to highlight the role of miR-92a in the modulation of NKT cells (Figure 7).

## 9. Resistance to Therapy

One of the greatest impediments in current GBM treatment is the resistance to radiation and chemotherapy. Cancer may be comparable to a complex organism trying to survive and thrive just like any other living organism. Its fast growth rate allows to natural select itself in a Darwinian manner, evading or defending itself of any harmful stimuli [159]. In turn, this creates extremely heterogeneous tumors and various interindividual GBM subtypes [160]. Plenty of biological mechanisms have been observed to be involved in radio- and chemoresistance: hypoxia and angiogenesis, oxidative stress and DNA damage repair, autophagy, Ca2+ homeostasis, apoptosis, proliferation, cell cycle, invasivity, and ATP-dependent drug efflux [159,161,162,163,164,165,166,167]. On top of that, the way this resistance is spread is just as impressive and complex. These mechanisms are influenced by miRNAs either directly by means of intercellular communication or indirectly by means of intracellular regulations of secretable factors that spread this resistance. 

TMZ is currently the first line chemotherapeutic agent in GBM therapy and acts by methylating DNA at the O6 and N7 positions of guanine and the N3 position of adenine. MGMT repairs the O6-methylguanine, and the base excision repair system (BER) repairs the N7 and N3 methylguanines [159]. 

Many miRNAs have been linked to therapy resistance in GBM cell lines, as well as in tumor samples [162]. The miR-181 family could be involved in MGMT posttranscriptional regulation. Zhang et al. have found that miR-181d was positively associated with TMZ response and patient survival. Consistent with this finding, transfecting miR-181d into the A1207 GBM cell line caused decreased MGMT mRNA and protein expression [168].

Many studies have described miR-21 as an oncomiR, its overexpression in the U87 glioma cell line being linked to protection against TMZ-induced apoptosis via decreased Bax/Bcl2 ratio and Cas3 activity [169]. miR-21 silencing was also shown to enhance the chemosensitivity of human GBM cells to sunitinib, temozolomide, doxorubicin, and paclitaxel [109,170,171,172]. A similar response has been observed for miR-221/222, their downregulation causing an increase in sensitivity to TMZ [173].

Also, TMZ-R GBM cells show an increased expression of Cx43, allowing the formation of connexons for the exchange of miRNAs across GJs [174]. Additionally, EGFR sits at the top of a downstream signaling cascade that controls basic functional properties of GBM cells, such as survival, cell proliferation, and migration [175]. The activation of EGFR leads to downstream signaling pathways such as the phosphorylation of AKT, MAPK, and JNK, also inducing Cx43 expression. It has been observed that TMZ favors EGFR activation, stimulating the transcription of Cx43 by MAPK-AP-1 [176]. Conversely, glioma-glioma GJs were shown to have anti-invasive effects, and particularly, miR-34a was demonstrated to inhibit GBM proliferation when transferred through glioma-glioma GJs [44,57]. However, TMZ-R clones acquire a distinctive set of intracellular miRNAs, and their transfer may contribute to the aggressive behavior. For instance, miR-1280, miR-1238, miR-938, and miR-423-5p are enriched in TMZ-R compared to their TMZ-S counterparts. miR-1238 is also highly expressed in TMZ-R-derived exosomes and confers resistance to adjacent cells by targeting the CAV1/EGFR pathway [176]. 

Stojcheva and collaborators found miR-138 was overexpressed in cell lines and recurrent GBM tissues and promotes proliferation of glioma cells [177]. Another upregulated miRNA in U87-resistant cells is miR-132, which inhibits the expression of tumor-suppressor candidate 3 (TUSC3), inducing the TMZ-R phenotype [178]. Still, a comprehensive signature of miRNAs in TMZ-R is yet to be identified, along with the possible transfer mechanisms. 

TNTs also confer high resistance to stress, efficient cellular proliferation, invasion, and efficient damage repair. Growth-associated protein 43 (*GAP43*), which normally helps neurons grow axons, is one critical gene that tumor cells use to extend these TNTs. Alongside *GAP43*, Cx43 is at the basis of the normal development and function of these structures. Interestingly enough, Cx43 builds gap junctions where these TNTs meet with neighboring cells, through which small molecules can pass. Damaged cells can also be “rescued” by nearby cells, providing them with nuclei and other resources by means of these microtubes [55]. Weil et al. demonstrated that these structures convey resistance to alkylating agents and might also be an explanation for the high relapse around surgical lesions [179].

Munoz et al. showed that TMZ-treated GBM cells caused an increase in miR-9 levels and an increase in EVs secretion [164]. miR-9 molecules have been shown to suppress mesenchymal differentiation of GBM cells. It has also been observed to mediate an increase in the drug efflux transporters MDR1 and ABCG2 which increases resistance to TMZ, thus spreading resistance to neighboring GBM cells [180,181].

Elucidating the mechanisms through which GBM cells use miRNAs to become resistant to conventional therapy is therefore essential. Reverse-engineering these mechanisms might lead to new and enhanced therapeutic approaches. Promising results have already been observed in mice. Interestingly, Bhaskaran et al. observed a synergic antitumor activity of miR-124, miR-137, and miR-128, leading to decreased GSCs proliferation and a better response to chemotherapy and radiation [182].

Big efforts are invested in understanding the vast regulatory pathways through which these molecules affect the response to therapy. However, it is still difficult to draw precise conclusions at this stage due to the high heterogeneity among studies, as well as the lack of replicability. More comprehensive research is still required before miRNAs can be viewed as a therapeutic option.

## 10. miRNAs as Biomarkers 

The early diagnosis of cancer can reduce the mortality, extend survival, and reduce treatment costs. While for some solid tumors, biomarkers such as CTCs (circulating tumor cells) can be used for diagnosis; for brain tumors, there is no established method at this time. However, since miRNAs have been shown to be deregulated in GBM, remarkable endeavors were made to establish a correlation between these RNAs and the presence and evolution of the disease [127]. Exosomes containing miRNA are released from the tumor and can be found in serum or CSF. Recent studies have shown that circulating miRNAs may provide a noninvasive diagnostic tool for glioblastoma (Table 2) [24,183,184,185]. 

Over the last years, several studies have tried to establish a miRNA panel that can be used to diagnose GBM. Among them, miR-21 is the most studied miRNA in GBM and received particular interest. However, since miR-21 dysregulation is present in other solid tumors and also nonmalignant diseases, searching for GBM-specific signatures in body fluids is still needed [198]. Nevertheless, considering there are approximately 2300 human miRNAs, finding a perfect panel of miRNAs will prove challenging [199].

Santangelo et al. studied three circulating miRNA (miR-21, miR-222, and miR-124-3p) and found a cumulative sensitivity of 84% for the diagnosis of glioblastoma. Additionally, miR-21, miR-222, and miR-124-3p were found to be significantly decreased after surgical resection in high-grade gliomas but not in low-grade gliomas [24]. Similarly, Ivo D’Urso et al. demonstrated that circulating miR-15b and miR-21 may be used as biomarkers in GBM, with 90% sensitivity and 100% specificity. miR-16 was shown to be able to discriminate between different grades of glioma [183].

The miRNA signature of GBM in blood samples includes miR-15b*, miR-21, miR-23a, miR-29, miR-133a, miR-150*, miR-197, miR-210, miR-454-3p, miR-497, and miR-548b-5p, these miRs being correlated with the WHO-grade and the survival [200]. In the CSF of GBM patients, 25 miRNAs were recently found to be dysregulated, upregulated miR-10b and miR-196b corresponding to a worse prognosis [25]. Another study showed nine miRNAs CSF signatures for the identification of GBM: upregulated miR-21, miR-193b, miR-218, miR-331, and miR-374a and downregulated miR-27b, miR-130b, miR-520f, and miR-548c [39].

Other studies compared miRNA levels in relation to the surgical resection. Yue et al. found miR-205 levels significantly increased after surgery and decreased after recurrence, suggesting miR-205 may have potential in monitoring tumor progression [192].

The role of circulating miRNAs will become more significant in the future of diagnostics, not only in GBM but in other cancers as well. Further research is mandatory in order to establish a reliable miRNA panel that will provide a minimally-invasive diagnostic tool that will allow early detection and treatment, monitoring therapeutic responses.

## 11. Conclusions

Despite therapeutic efforts, GBM remains one of the most lethal tumors. Having an extremely heterogeneous organization, GBM presents a multitude of cellular subpopulations organized both following Darwinian and hierarchical models. miRNAs represent a fundamental mechanism of post-transcriptional control, highly involved in GBM cell-cell communication. Within the GBM microenvironment, miRNA molecules seem to circulate in specific directions, mediating interactions between GBM cells, astrocytes, and ECs.

One of the most important miRNAs is miR-5096. Multiple independent studies revealed that it is transferred by the GBM to ECs and astrocytes. Therefore, it plays a major role in intercellular communication, influencing both the angiogenic properties of ECs and probably the proliferation of activated astrocytes.

miR-21 present in exosomes derived from GBM influences the whole tumor microenvironment. In the ECs context, miR-21 favors angiogenesis through the agency of VEGFR2. To a greater extent, miR-21 plays a part in the response to antiangiogenic therapy. The administration of bevacizumab was proven to enhance the serum levels of miR-21. Of significant importance is the bidirectional exchange of miR-21 between GBM and the macrophages responsible for maintaining both cellular subpopulations in an anti-inflammatory, pro-tumorigenic STAT3 phenotype. It should be taken into consideration that this mechanism has not been fully explained. So far, only unidirectional studies on GBM and macrophages were completed. The use of GBM cells and fluorescent marked macrophages, along with the determination of pSTAT3 in co-cultures, could better illustrate a bidirectional STAT3/miR-21-mediated relationship, arguing for the use of STAT3 inhibitors in tumor microenvironment modulation.

The interaction between GBM and astrocytes requires additional studies in order to properly determine and clarify the roles played by miR-10b, miR-5096, mi-R-709, and miR-19a. Additionally, miR-219-5p, mi-R-219-2-3p, and miR-338-3p were shown to contribute to oligodendrocytes’ differentiation. However, taking into account the macrophages and microglia augmentation at the border niche, along with the effects these miRNAs had in vitro, a potential transfer between oligodendrocytes and immune cells should be investigated. Considering the miRNAs’ particular signatures in GCSs, it is absolutely essential to map the mRNAs targeted by them and identify the pathways through which they modulate the secretome in order to acquire treatment resistance.

The main problem arises from the current data that results from studies performed in vitro on monocellular cultures that do not fully reflect the tumor heterogeneity, architecture, and microenvironment. We encourage the use of more complex models: 3D cultures, brain slices, and patient-derived organoids. The deregulations of numerous miRNAs were confirmed in patients, along with IHC evidence regarding intercellular relationships in vivo. Still, a more sensitive and specific panel of miRNAs in body fluids should be researched. In addition, more accurate models that recreate the TME could offer a more precise perspective regarding the concept of microglia/macrophages activation. Attempts to reeducate these cells to the M1-like phenotype could lead to unwanted consequences regarding the importance of TNF-α in maintaining malignancy. Outdated theories of the M1-to-M2 switch should be adapted in the in vivo reality in order to investigate complex patterns in such conditions.

The trend in GBM research is heading towards molecular therapies aimed at tumorigenesis key points. However, the interindividual variability of GBM subtypes and the diversity in the activation of relevant signaling pathways will reorient research towards personalized medicine. In this context, a more exact mapping of the network formed by miRNA in cell-cell communication is a goal that could open new paths to a more efficient treatment.

Numerous mechanisms are not yet understood, and this review attempted to highlight the gaps in the literature and possible directions of future research. Understanding these communication mechanisms mediated by miRNAs and their spatial distribution will lead to novel molecular therapies targeting miRNA-based signaling pathways. The identification of miRNAs’ involvement in cell-cell communication in glioblastoma is the key to understanding the tumor biology for a better patient stratification and new individual-based therapeutic approaches.

## Figures and Tables

**Table 1:** Principal interactions between glioblastoma (GBM) cells and normal cell populations in the tumor microenvironment. EV: extracellular vesicle, EC: endothelial cell, GSC: glioma stem cell, and GA-hMSC: glioma-associated mesenchymal stem cell; (↑ increased; ↓ decreased).

Intercellular Transfer	miRNA	Mechanism of Transportation	Effect	Reference
GBM ↔ GBM	miR-5096	Gap junctions	Invasion ↓	[56]
GBM → Astrocyte	miR-5096 miR-4519	Gap junctions	Invasion ↑	[57]
EC → GBM	miR-145-5p	Gap junctions	Proliferation ↓	[46]
GBM → EC	miR-5096	Gap junctions	Invasion ↑ Angiogenesis ↑	[46]
GSC → EC	miR-21	Exosomes	Angiogenesis ↑	[58]
GA-hMSC → GSC	miR-1587 miR-3620-5p	Exosomes	Proliferation ↑	[59]
GBM → Microglia	miR-21 miR-451	EVs	Proliferation ↑	[60]
GBM ↔ Macrophage	miR-21	EVs	Invasion ↑ Proliferation ↑ Angiogenesis ↑ Resistance ↑	[61,62]

**Table 2:** Deregulated miRNAs in body fluids. CSF: cerebrospinal fluid. (↑ increased; ↓ decreased).

Year	No. of Cases	No. of Controls	Body Fluid	Technique	miRNAs	Reference
2011	20	20	Blood	qRT-PCR	↑miR-128	[186]
					↑miR-342.3p	
2012	10	10	Plasma	qRT-PCR	↑miR-21	[187]
					↑miR-128	
					↑miR-343-3p	
2013	133	80	Serum	qRT-PCR	↓miR-15b	[188]
					↓miR-23a	
					↓miR-133a	
					↓miR-150	
					↓miR-197	
					↓miR-497	
					↓miR-548-5p	
2014	75	55	Serum	qRT-PCR	↑miR-320	[189]
					↑miR-574-3p	
					↑RNU6-1	
2015	30	30	Plasma	qRT-PCR	↑miR-15b	[183]
					↑miR-21	
					↓miR-16	
2015	126	40	Serum	qRT-PCR	↑miR-210	[190]
2016	112	54	Plasma	qRT-PCR	↑miR-182	[184]
2016	50	51	Plasma	qRT-PCR	↑miR-221/222	[191]
2016	64	45	Serum	qRT-PCR	↓miR-205	[192]
2016	15	10	Serum	qRT-PCR	↓miR-497	[193]
					↓miR-125b	
2017	111	84	CSF	qRT-PCR	↑miR-21	[39]
					↑miR-218	
					↑miR-193b	
					↑miR-331	
					↑miR-374	
					↓miR-548c	
					↓miR-520f	
					↓miR-27b	
					↓miR-130b	
2017	100	50	Serum	q-RT-PCR	↓miR-376a	[194]
					↓miR-376b	
					↓miR-376c	
2018	60	43	Serum	qRT-PCR	↑miR-301a	[195]
2018	100	30	Serum	qRT-PCR	↑miR-21	[24]
					↑miR-222	
					↑miR-124-3p	
2018	41	21	CSF	qRT-PCR	↑miR-10b	[25,196]
					↑miR-196b	
2019	107	80	Serum	qRT-PCR	↓miR-29b	[197]
2019	95	60	Serum	qRT-PCR	↓miR-100	[185]
